# The impact of Clinical Trials Radiographers on set-up and recruitment to radiotherapy trials

**DOI:** 10.1016/j.tipsro.2025.100309

**Published:** 2025-04-04

**Authors:** Donna Caldwell, Aileen Duffton, Chloe Wilkinson

**Affiliations:** a The Beatson West of Scotland Cancer Centre, Glasgow, UK; b Institute of Cancer Sciences, University of Glasgow, UK

**Keywords:** Clinical trial, Therapeutic Radiographer, Clinical Trial Radiographer, Clinical trial set-up, Clinical trial recruitment

## Abstract

**Background:**

The primary role of the Clinical Trials Radiographer (CTR) within the UK workforce is the set-up and initiation of new trials, and recruitment of patients. There is very little published evidence to indicate the impact and value of the CTR. The aim of this study is to describe the evolving role of the CTR and to quantify impact on set-up, and recruitment to Radiotherapy (RT) clinical trials.

**Results:**

The number of trials approved and opened annually, number of open studies within the portfolio, and number of patients recruited have all increased alongside the number of CTR hours.

**Conclusion:**

The data provides quantitative evidence to support the impact of the CTR role and its value in a RT department’s research infrastructure. This reinforces the need to consider the CTR position in long term funding and future workforce planning.

## Highlights

• Evolving role of the Clinical Trials Radiographers.
• Demonstrating the positive impact of Clinical Trials Radiographers.
• Clinical Trials Radiographers increase the number of studies opened.
• Increased Trial availability improves access to Radiotherapy trials for patients.

## Introduction

Radiotherapy (RT) plays a vital role in the treatment of cancer, being used to cure localised disease, palliate symptoms, and control disease in incurable cancers. It is received by approximately half of all patients during their treatment journey, with around 40 % of patients cured having undergone RT [1,2]. A key driving force behind improved cancer care and reduced side effects has been the successful conduct and dissemination of extensive international RT clinical trials, which has provided data for evidence-based changes in practice [[3], [4], [5], [6], [7]]. There should be external independent radiotherapy trial quality assurance (RTQA) to ensure treatment in a trial is introduced safely and consistently across participating sites through protocol compliance [8,9].

Patient pathways can encounter several variations from initial referral, right through to delivery of treatment [10]. In the context of a clinical trial, RT delivery requires rigorous QA to ensure trial outcomes are due to the merit of the study rather than any discrepancies in treatment delivery [11,12]. Independent third-party bodies undertake RTQA, which is in place to provide a complete and comprehensive review of all aspects of the RT pathway [13]. Increasing complexity of RT planning and delivery requires more sophisticated QA which can lengthen trial set-up and make implementation considerably more resource intensive. Streamlining of RT trial QA reduces the QA requirements of subsequent similar trials, which can be available depending on QA provider and previous trial participation. Several studies demonstrate the benefit of clinical trial QA in both the outlining and planning phases of the patient’s RT pathway [[14], [15], [16], [17], [18]].

The absence of dedicated RT research roles has been recognised as a recurring issue and barrier to clinical trial set-up [19]. Clinical Trial Radiographer (CTR) posts have been implemented across the UK to underpin trial work. The Research and Clinical Trials Therapeutic Radiographer Network, a recognised College of Radiographers Special Interest Group, recorded a substantial increase in membership between 2017 and 2020, reflecting the growing number of these roles. However, variation in the activities performed by CTRs between centres presents challenges in assessing the impact of the role [20]. While anecdotal evidence supports the value of the CTR role, auditable evidence is essential for securing long term funding and informing future planning [21].

A survey conducted by Taylor and Shuttleworth (2021) identified the primary roles of UK based CTRs as the set-up and initiation of new trials, carried out by 53 %; and participating in patient recruitment to clinical trials (42.5 %) [22]. Although this survey outlined the size, structure and scope of the UK CTR workforce, there is a lack of published quantitative evidence to show the impact such roles have had.

The CTR position has been established in our department since 2013 and we now have over a decade of experience to bridge the evidence gap. Specifically, this work aims to describe the evolving role of the CTR and quantify the impact on set-up, and recruitment to RT clinical trials.

## Methodology

### Role tasks and role development

The evolving role of the CTR was described by 2 CTRs, each with >6 years’ experience in the role. CTRs working at the department reviewed the 2017 job description to identify tasks that influenced the opening of, or recruitment to RT clinical trials. In addition, they provided updated task descriptions as of the 31st December 2022, and provided rationale for the changes, including descriptors of efficiency improvements and service benefit.

### Number of open/recruiting RT trials

EDGE v2, clinical research management system (a purpose-built programme designed to track and manage studies including participant recruitment) [23], had been used by CTRs to maintain an up-to-date registry of all trials involving RT and requiring CTR support.

Radiotherapy Management Group (RMG) is the local management group with representatives from radiotherapy, physics and clinical oncology. This includes heads of each department, research leads and the site senior management team. Departmental set-up can only commence on a study once approval has been granted by this group.

A report was generated from EDGE v2 to include RMG approval date, trial opened and closed dates and recruitment figures for all relevant studies. The report underwent cross-verification with local records to identify any missing trials, thereby ensuring data completeness.

The analysis included data from 2012 to 2022 which was sub-divided by RMG approval year. Duration to open a study was measured as the time between RMG approval date and open date. The mean duration to open a study was also calculated for each year. 2012 was included to provide a baseline prior to the creation of the CTR position.

Annual trial recruitment and CTR whole time equivalent (WTE) positions were also calculated for each calendar year.

### Covid adjustment

All local trial related work was suspended for 4 months starting April 2020. To adjust for COVID-related trial suspension, mean ‘COVID adjusted’ durations were calculated by subtracting 120 days for trials in set-up over that period. Individual trials restarted following a risk assessment, therefore 120 days was the minimum applicable suspension for each trial.

## Results

### Role tasks and role development

The evolving role of the CTR is summarised in Table 1.

**Table 1:** CTR Tasks that affect opening of/recruitment to clinical trials.

CTR Tasks That Affect Opening of/Recruitment to Clinical Trials		
Original Task (Job description 2017)	Task Evolution (by 31st December 2022)	Efficiency Improvement/Service Benefit
Review proposed radiotherapy clinical trials and evaluate radiotherapy resource implications at Clinical Trials Executive Committee (CTEC)^a^	Complete and submit CTEC form on behalf of Principle Investigator (PI). PI review is required prior to final submission. Present radiotherapy clinical trials on behalf of PI if required.	A single point of contact between CTEC and the radiotherapy department (radiotherapy and physics) streamlines communication. PI commitments can make paperwork completion and meeting attendance difficult. Reassigning PI tasks reduces delays in CTEC approval timeline.
Radiotherapy Management Group (RMG)^b^ attendance when a radiotherapy clinical trial is on the agenda for approval to discuss resource implications and answer queries.	Redesign of the RMG Clinical Trial Submission Process. Complete and submit RMG form on behalf of PI. PI review is required prior to final submission. Provide monthly clinical trial portfolio and recruitment overview as a rolling agenda item.	Redesign of the submission process means radiotherapy and physics departments review trials prior to the RMG meeting, issues are addressed in advance, reducing RMG approval timeline. PI commitments can make paperwork completion and meeting attendance difficult. Reassigning PI tasks reduces delays in RMG approval timeline. RMG have oversight of trial portfolio, influencing decision making on trial support.
Act as a specialist resource to inform ethics submissions.	Complete Integrated Research Application System (IRAS) submissions for specific international clinical trials.	Completing this process allows important radiotherapy trials to open that do not have clinical trial unit (CTU) support to complete this task. These trials would have failed to open or been significantly delayed without this intervention.
N/A	Act as a Clinical Trial Coordinator/Data Manager for data collection clinical trials.	Taking on this process allowed important radiotherapy trials to open that did not have CTU support. These trials would have failed to open or been significantly delayed without this addition to the role.
Contribute to service development.	Innovate service development by creation and implementation of process and practice changes.	Creation of a local multidisciplinary carepath within the radiotherapy planning system for trial QA has improved efficiency with: • streamlined communication • reduced delays in completion of pre-trial QA • prompt on-trial QA returns • improved data collection
Contribute to the radiotherapy treatment information required for trial QA.	Coordination of all clinical trial QA requirements.	A single point of contact between the QA provider and the radiotherapy department (radiotherapy and physics) has resulted in streamlined communication.
N/A	Submission of retrospective/data collection radiotherapy plans	The return of retrospective/data collection radiotherapy plans has improved. This work is also now recorded as all trial activity should be.
Use EDGE to record recruits to trials.	Develop the use of EDGE in radiotherapy trials.	Updated workflows within EDGE communicate trial QA requirements and set-up progress to the wider trial team network, streamlining communication. Forms are created and completed in EDGE to provide radiotherapy data to the CTU. Streamlining communication and providing prompt return of data needed for case report form completion.

a Clinical Trials Executive Committee (CTEC) is our hospital’s internal steering committee, it reviews all studies that involve patient contact at our department with representatives from service disciplines, trial unit, clinicians and PPI.

b Radiotherapy Management Group (RMG) is our local radiotherapy department’s management group with representatives from radiotherapy, physics and clinical oncology.

### Trial set-up and trial recruitment

**Fig. 1:**
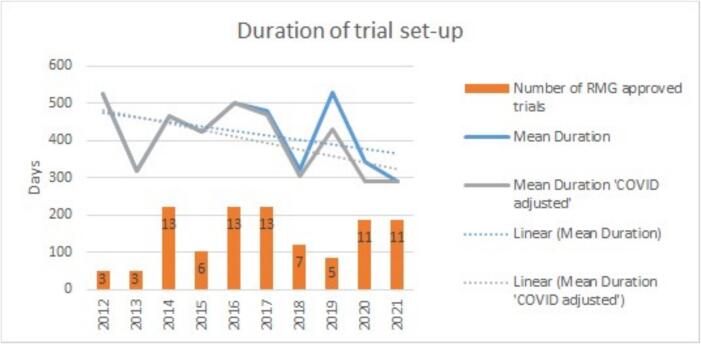
Number of trials receiving RMG approval per annum (number displayed on annual columns), mean duration of trial set-up in days, with and without COVID adjustment.

One hundred RT involved trials that utilised CTR resources were identified during the inclusion period. The annual number of RMG approved trials varied between 3 and 13. The mean time taken to open a trial decreased by 44.8 % from 525.3 days in 2012 to 290 days in 2021 (Fig. 1).

**Fig. 2:**
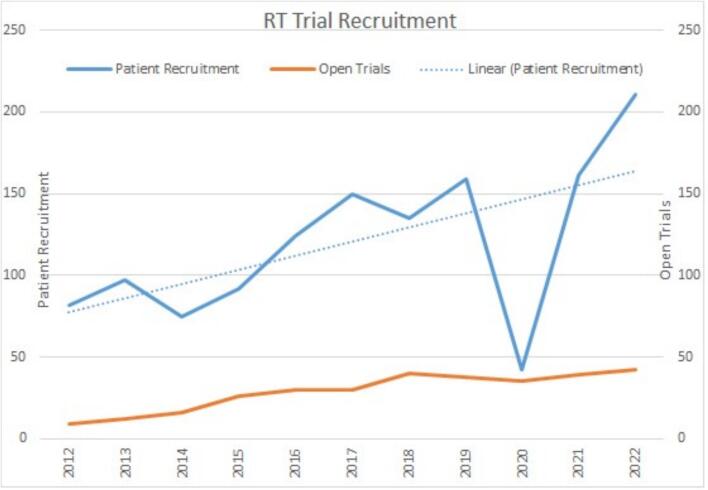
Annual number of recruiting radiotherapy trials and corresponding patient recruitment.

The number of open studies in the local departmental portfolio increased from 9 in 2012 to 42 in 2022, during which period annual patient recruitment increased from 82 to 211 (157.3 %) (Fig. 2). Patient recruitment declined in 2020 to 42 due to the suspension of all trial activities, including patient recruitment, related to the COVID 19 pandemic.

**Fig. 3:**
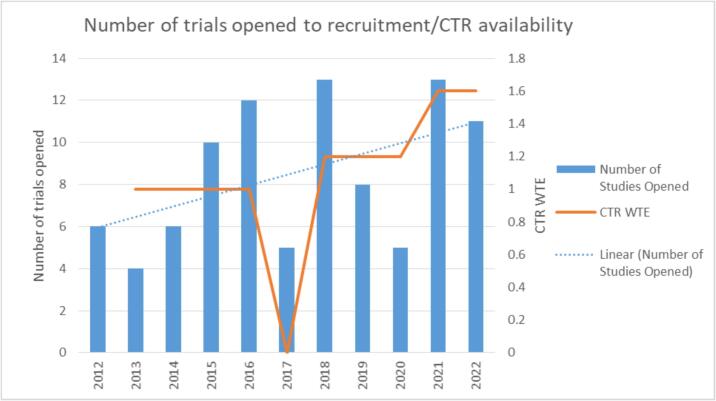
Number (n) of new trials opened to recruitment annually, shown alongside Clinical Trial Radiographer whole time equivalent (WTE).

The annual number of trials opened varied between 4 and 13 an overall increase over time from 6 in 2012 to 11 in 2022 (Fig. 3). Following an increase in 2015 and 2016, the number of studies reduced to 5 in 2017, only 2 of these 5 studies opened during the period where 0 CTR hours were supporting trial setup i.e. during a 9-month vacancy period when the CTR position was unfilled. In 2018 the number of studies opened increased to 13, with CTR support increasing by 7.5 h per week. In 2020, the number of trials opened reduced to 5, coinciding with a COVID-related suspension of trial work.

## Discussion

The aim of this work was to describe the role of the CTR and quantify impact on the opening and recruitment to RT trials. We have successfully outlined the evolving CTR role in our department and have presented quantitative data, obtained from our clinical research management system, which demonstrates the impact. To be more widely applicable across the UK we focused on two prominent themes within the UK CTR workforce scope of practice i.e. trial set-up and initiation and trial recruitment [22].

The number of trials approved and opened annually, number of open studies within the portfolio, and number of patients recruited have all increased alongside the number of WTE CTR positions, where CTR hours have increased from 1 WTE in 2013 to 1.6 in 2021.

### Role tasks and role development

The CTR role was introduced in our department to facilitate the set-up of RT clinical trials, consistent with the majority of the CTR UK workforce [22]. By reviewing our CTR tasks and their evolution (Table 1) we reflected on changes that have occurred between the 2017 job description and current practice as of end of year 2022. While some adaptation occurred gradually over time, other changes were implemented with deliberate intention. The review of all elements enabled us to identify efficiency improvements, service benefits as a result of the changes, and highlight recurring themes.

The process of opening RT clinical trials is truly multi-disciplinary, including radiographers, physicists, dosimetrists and clinical oncologists, supported by non-clinical trial unit and research and innovation (R&I) staff. Moreover, depending on the nature of the trial, there is coordination with other services such as nursing, pharmacy and diagnostics. This work only discusses the CTR role in detail, however the contribution of the MDT is acknowledged.

### Overcoming barriers

Identifying bottlenecks in the set-up process has led to reassigning duties to the CTR conventionally undertaken by the PI, traditionally a Consultant Clinical Oncologist. While not limited to this, it includes completing regulatory paperwork for governance and presenting at local approval meetings. Breaking from tradition, where appropriate, the CTR has been PI and encourages and supports other specialist Radiographers within the department to also become PIs on both internal local studies and externally funded multicentre studies. As highlighted in the 2022 RCR census this shift is becoming increasingly necessary as consultants’ workloads expand, leaving them without the capacity to fully engage in clinical research [24].

The CTR task review highlighted involvement in activities, such as IRAS form completion and data management that are usually undertaken by clinicians, project managers and clinical trial coordinators. Performing these tasks and broadening the scope of the CTR role has enabled trials to be opened that otherwise would have lacked resources or faced significant delays.

Two aims of our health board R&I strategy are to increase the number of multi-disciplinary PIs and to increase the number of high-quality, impactful research studies and innovation projects [25]. These examples demonstrate how the diverse expertise of the CTR enhances the multi-disciplinary trial set-up process and can be utilised to achieve the organisation’s strategic aims.

### Communication

As RT is a regional service, communication is required across the main RT site and satellite centre; multiple referring health boards; local trial teams; sponsor teams and QA providers. The more stakeholders involved, the more complex information flow becomes [26]. A major benefit of the CTR role is having a single point of contact for the network and external bodies. This streamlined communication results in timely responses to queries, facilitates tracking and prompting of local submissions and dissemination of relevant feedback, which avoids missed submission deadlines and reduces set-up delays.

Creating workflows and attributes within EDGE allows the progress of trial set-up to be tracked by relevant staff across the network of trial sites and RT trial data to be provided promptly for case report form completion. Implementation of task based carepaths within the RT planning system allows staff to follow a process similar to that of off-trial clinical work whilst ensuring necessary additional trial information is obtained. Utilising these electronic work spaces and process automation has reduced email traffic and delays waiting for instruction because work is allocated proactively rather than reactively.

By minimising barriers and improving communication, mean duration to open a study has reduced by 44.8 %.

### Recruitment

The considerable involvement of RT in the patient treatment journey is not reflected in RT clinical trial investment. RT trials account for a minority (5.3 %) of all oncological trials and only 5.8 % of commercially sponsored trials [27]. Trial activity and recruitment are important measures for securing funding for RT research, an area already underfunded [28,29].

Like a large proportion of the UK CTR workforce, local CTRs enhance recruitment in a number of ways, such as identifying potential patients at MDTs and screening patient lists. Patients are contacted by CTRs to gauge their interest in participation and answer trial related queries. Doing this and recruiting patients to non-interventional, translational or data collection studies has been successful in increasing recruitment figures, another R&I strategy aim [25] and a significant outcome when 96 % of heads of services said workforce shortages are restricting clinical trial recruitment [24].

CTR contribution to patient recruitment is two-fold, by the CTR recruiting or assisting others to recruit and then by creating and supporting a larger trial portfolio containing a broader study type to capture a wider scope of eligible patients. The CTRs facilitate the opening of new trials allowing the continued recruitment of patients by PIs and delegated tumour specific staff. During the 10 year timeframe analysed, the number of trials available to recruit patients into increased from 9 in 2012 to 42 in 2022 and annual patient recruitment increased by 157 %.

### Other contributing factors

Since the creation of the post in 2013, our department’s CTR WTE hours have increased by 60 %, driven by role reflection and workload projection. Over this same time the number of trials opening to recruitment annually has increased. Pinpointing one exact reason for the increased number of trials being opened is difficult, but it is hoped that the enhanced guidance and support provided by the CTR has made the set-up process less daunting and raised the profile of clinical trials within the RT department. Furthermore, our data indicated that the lack of CTR support in 2017 considerably reduced the number of trials opening for recruitment. The CTR post was vacant for 9 months of that year, during which only two trials opened. The remaining three trials were initiated in the final three months of the year once the CTR position was filled.

Independent third-party RT trial QA can be resource intensive. However, streamlining QA, where participation in a previous trial reduces the QA requirements of subsequent similar trials, positively impacts trial set-up duration [9,15,17]. Meaning initial resource investment can result in reduced future workload, which would imply a reduction in set-up time. However, QA streamlining does not apply to all trials, nor is it available across all providers due to more complex QA being required [18].

### Strengths and limitations

Our department provides radiotherapy across a network of trial sites covering multiple health boards. The clinical research management system enables an up-to-date registry of all trials with a RT component and requiring CTR input. By utilising these systems, we were able to authenticate the data and ensure completeness.

Whilst the CTR role has undoubtedly contributed to reducing trial set-up duration and overcoming bottlenecks, it is ultimately a multi-disciplinary process. The changes driven by the CTR role alone, albeit valuable are challenging to quantify in isolation. In particular, Clinical Oncologist and Medical Physicist resource are fundamental in RT trial set-up but other resources such as pharmacy and nursing can also make substantial contributions. The Academy of Medical Sciences report, Transforming health through innovation: Integrating the NHS and academia [30] recommends dedicated time for research across the healthcare workforce. The CTR role within our department is dedicated to RT clinical trials but unfortunately this protected time is not replicated across all staff groups, or across the UK CTR workforce [20,31]. When other staff groups lack dedicated time or resources for clinical trials, set-up can be prolonged, and the CTR has minimal ability to circumnavigate this.

## Conclusion

Results demonstrate the contribution and the benefits of the CTR post in opening an increased number of studies, in a reduced timeframe. With increased trials available and a streamlined opening process, elongating the recruitment period, there is improved access to RT clinical trials for patients.

A diverse RT trial portfolio is demanding. This data provides quantitative evidence to support the impact of the CTR role and its value in a RT department’s research infrastructure. This reinforces the need to consider the CTR position in long term funding and future workforce planning.

## Declaration of competing interest

The authors declare the following financial interests/personal relationships which may be considered as potential competing interests: Author Aileen Duffton is an Associate Editor but was not involved in the editorial review or the decision to publish this article.

The author is an Editorial Board Member/Editor-in-Chief/Associate Editor/Guest Editor for this journal and was not involved in the editorial review or the decision to publish this article.
